# A Feasibility Study of the Addition of STEPPS in Outpatients With Bipolar Disorder and Comorbid Borderline Personality Features: Promises and Pitfalls

**DOI:** 10.3389/fpsyt.2021.725381

**Published:** 2021-11-11

**Authors:** Georg Riemann, Melissa Chrispijn, Nadine Weisscher, Eline Regeer, Ralph W. Kupka

**Affiliations:** ^1^Department of Applied Psychology, Saxion University of Applied Sciences, Deventer, Netherlands; ^2^Dimence Mental Health, Center for Bipolar Disorders, Deventer, Netherlands; ^3^Geestelijke Gezondheids Zorg (GGZ) Heuvelrug, Center for Mental Health, Driebergen, Netherlands; ^4^Center for Bipolar Disorders, Altrecht Institute for Mental Health Care, Utrecht, Netherlands; ^5^Amsterdam University Medical Center (UMC), Department of Psychiatry, VU University, Amsterdam, Netherlands; ^6^Geestelijke Gezondheids Zorg (GGZ) InGeest, Center for Mental Health Care, Amsterdam, Netherlands

**Keywords:** bipolar disorder, borderline personality features, STEPPS group therapy, comorbidity, prevalence

## Abstract

**Background:** Pharmacotherapy is a cornerstone in bipolar disorder (BD) treatment whereas borderline personality disorder (BPD) is treated primarily with psychotherapy. Given the overlap in symptomatology, patients with BD may benefit from psychotherapy designed for BPD.

**Aims:** This paper reports the findings of a non-controlled open feasibility study of STEPPS training in patients with BD and borderline personality features (BPF).

**Methods:** Outpatients with BD were screened for BPD, and if positive interviewed with SCID-II. Patients with at least three BPF, always including impulsivity and anger burst, were included in the intervention study. Severity of BD and BPD and quality of life were assessed. Descriptive statistics were performed.

**Results:** Of 111 patients with BD 49.5% also screened positive on BPD according to PDQ-4+, and 52.3% of these had BPD according to SCID-II. Very few participants entered the intervention study, and only nine patients completed STEPPS. Descriptive statistics showed improvement on all outcome variables post treatment, but no longer at 6-month follow up. We reflect on the potential reasons for the failed inclusion.

**Conclusion:** Features of BPD were highly prevalent in patients with BD. Still, recruiting patients for a psychological treatment originally designed for BPD proved to be difficult. Feedback of participants suggests that the association of STEPPS with “borderline” had an aversive effect, which may have caused limited inclusion for screening and subsequent drop-out for the treatment. Therefore, STEPPS should be adapted for BD to be an acceptable treatment option.

**Clinical Trial Registration:** www.ClinicalTrials.gov/3856, identifier: NTR4016.

## Introduction

Bipolar disorder (BD) is a mental illness characterized by recurrent manic, hypomanic and depressive episodes separated by intervals of variable duration with relatively few symptoms (1). Most common subtypes are Bipolar-I disorder (BD-I; depressive and manic episodes) and Bipolar-II disorder (BD-II; depressive and hypomanic episodes only). Overall, depressive symptoms and episodes are more common than (hypo)mania (2–4). A majority of BD patients experience a comorbid condition (5) such as substance abuse (6, 7), anxiety disorder (8), or attention-deficit hyperactivity disorder (9). Co-occurrence of multiple disorders can lead to higher rates of suicidality and less response to pharmacological treatment (10). There is also evidence that the presence of comorbid personality disorder (PD) has an unfavorable impact on the course of BD. Post et al. (11) reported a close relationship between PD as measured by the self-rated 99-item Personality Disorder Questionnaire (PDQ-4) and poor prognosis factors of the course of BD such as history of child abuse, early age of onset, anxiety disorder comorbidity, rapid cycling course, and a high number of previous episodes. A longitudinal prospective study among respondents with a bipolar spectrum disorder (12) found that PD symptom severity predicted shorter time to hypomania and major depression (both particular in case of PD cluster B), and conversion to BD-I disorder (particular in case of PD cluster C) (13). Prevalence of PD in BD is estimated between 30 and 40%, which is significantly higher than in the general population (14). This concerns particularly PD from the B and C cluster, and especially borderline personality disorder (BPD) (15, 16). A systematic review and meta-analysis included 42 papers considering BPD in BD as well as BD in BPD concluded that up to 21.6% of BD samples have comorbid BPD with higher rates in BD-II than in BD-I (17). Although a comprehensive literature review (18) concluded that BD and BPD can be distinguished by comparing their phenomenology, etiology, family history, biological studies, outcome and response to medication in clinical practice the differential diagnosis between BD and BPD can be difficult given shared core characteristics of mood instability, emotional dysregulation and impulsivity (19). A study based on self-report measure showed great impairment in emotional regulation in both BD and BPD, but maladaptive strategies were greatest in comorbid than BPD and BD groups (20). A study by our group confirmed that affective instability, impulsivity, and self-mutilation/suicidality were symptoms associated with both rapid cycling BD and BPD (21). However, BD and BPD require different treatment approaches. Pharmacotherapy is a cornerstone for acute and maintenance treatment of BD (22–26), whereas BPD is primarily treated with psychological interventions. Evidence for the efficacy of psychotherapy in BD is less consistent. A recent review included 35 reports of 28 randomized controlled trials between 1995 and 2013 investigating individual or group psychosocial interventions for adults with BD (27), and found that psychotherapies, in combination with medication, show advantages over medication alone on measures of symptom burden and risk of relapse. Psychotherapeutic interventions that have demonstrated some prophylactic efficacy as an adjunct to medication in BD are individual (28) and group psychoeducation (29), cognitive behavioral therapy (CBT) (30, 31), Family-focused therapy (FFT) (32), and Interpersonal and Social Rhythm Therapy (IPSRT) (33). A meta-analysis including 55 trials with 6010 participants found that there is some evidence that psychological interventions are effective for people with BD, but further research is needed (34). Despite a high prevalence of comorbidity between BD and PD, and evidence that PD traits are associated with an adverse course of BD (11, 15, 35–37), there are no specific treatment recommendations for patients with comorbid PD. Given the overlapping phenomena in BD and BDP, we hypothesized that a treatment that is designed to improve emotion regulation in BPD, such as Systems Training for Emotional Predictability and Problem Solving (STEPPS) (38) could also be effective in BD and improve its overall course.

### Aims of the Study

The original aim of the study was to evaluate the effectiveness of Systems Training for Emotional Predictability and Problem Solving (STEPPS) in BD with comorbid BPF in a Randomized Controlled Trial (RCT). The original RCT study protocol is described elsewhere (39).

Given the very low inclusion of patients, for reasons that will be discussed later, we restricted our study to an open feasibility study in which we explored whether STEPPS would improve BD and BPD symptoms and quality of life immediately after the training and at 6 months follow-up. This brief report describes these explorative results and reflects on factors that may have limited the inclusion of sufficient participants to conduct the originally designed RCT.

## Methods

### Participants

Participants were recruited from four non-academic outpatient clinics for BD in the Netherlands. All participants received TAU for BD, consisting of guideline-recommended pharmacotherapy, psychoeducation and supportive counseling. Patients aged between 21 and 65 years who met the DSM-5 diagnosis criteria of BD-I, BD-II or BD-NOS, as confirmed during clinical interview by the treating psychiatrist, were screened for BPD with the Personality Disorders Questionnaire (PDQ-4+) (40). In case of a positive screening for BPD, participants received a semi-structured diagnostic interview (see section Materials and Procedure). Inclusion criteria for the treatment part of the study were having BD as well as at least three of the nine DSM-5 (1). BPF, always including impulsivity and anger burst, since these are a main focus in STEPPS. Affective instability was not obligatory given the overlap with BD. Exclusion criteria were severe depressive and manic symptoms, being currently treated for substance abuse, currently receiving another formal psychotherapy, having received STEPPS within the last 2 years, being unable to comply with the STEPPS protocol, and being unable to understand the Dutch language. The treating clinician determined whether a patient was potentially suitable for the study.

### Materials and Procedure

BD patients were informed about the two parts (screening and potential participation in the treatment intervention) of the study by their treating clinician. Patients who were interested were referred to the investigators and received both written and oral information about the study. Prior to participation patients signed an informed consent form. Included patients were screened for personality disorder traits by completing PDQ-4+ (40). In case of positive BPD screening patients were invited for a session including three semi-structured diagnostic interviews in order to confirm diagnoses of BD [Mini International Neuropsychiatric Interview, MINI Plus (41)], having BPF as defined for this study [Structured Clinical Interview for DSM-IV axis II, SCID-II (42)] and the severity of these BPF according to the Borderline Personality Disorder Severity (BPDSI) (43). Eligible patients then completed the Brief Symptom Inventory (BSI) (44) at baseline for severity of general psychological symptoms, and the Inventory of Depressive Symptomatology (IDS-SR) (45) for severity of depressive symptoms. During the STEPPS treatment, severity of borderline symptoms was assessed with the Borderline Personality Disorder Checklist (BPDC-47) (46) and Personality Assessment Inventory—Borderline Features (PAI-BOR) (47). Quality of life was measured with the Outcome Questionnaire (OQ-45) (48) and the World Health Organization Quality of Life short version (WHOQoL Bref) (49). Patient empowerment was assessed with the Patient Activation Measure (PAM) (50). Follow up measures were immediately after completing the 20-week treatment phase and at 6 months follow-up.

### STEPPS Treatment

STEPPS is a CBT based group program primarily designed for BPD patients to improve skills for emotion regulation. The underlying assumption is that BPD is characterized by impairment of the individual's internal ability to regulate emotional intensity. The aim of the training is to gain skills to manage emotions and behavior related problems. It is delivered by two trainers and consists of 20 weekly sessions of 2.5 h. STEPPS includes four parts: psycho-education, emotion regulation skills, behavioral skills, and emotion handling plan. Various studies demonstrated the efficacy of STEPPS in BPD (51–54). STEPPS is included in the Dutch guidelines for treatment in BPD (55). The current treatment study was carried out in regular STEPSS treatment groups at four sites, thus also including patients not having BD, and not participating in the study protocol. All psychotherapists were trained and qualified in STEPPS.

### Statistical Methods

Since the final sample size (*n* = 9) was too small to meet the assumptions of an analysis of variances, only descriptive statistics were conducted in order to explore the impact of the STEPPS on BD and BPD symptoms and quality of life, and these results are presented visually.

### Ethical Review and Registration

The originally designed study as well as the amendment covering the changes from RCT to open study as described above were reviewed and approved by the Ethics Committee of the VU University Medical Center in Amsterdam, The Netherlands (registration number: 2012/470). It is registered in www.trialregister.nl (trial ID: NTR4016).

## Results

### Demographics and Selection for Treatment

In total 111 patients completed the PDQ-4+. Patient's baseline demographic and clinical characteristics (age, gender, BD diagnosis) are summarized in Table 1. Age ranged from 19 to 67 years, 51.3% was married or living together, and median educational level was 3 (range 1–5). The process of selecting eligible patients for the treatment study is also shown in Table 1. In every stage of the study (screening for BPD; interviewing for BPD/BPF; entering treatment) patients were excluded from further study according to the protocol, or dropped out by not responding to the invitation after screening (*n* = 11) or by declining to participate in the treatment part of the study (*n* = 12).

**Table 1:** Demographic and clinical characteristics of patients with bipolar disorder in various phases of the study.

**Study phase**	**n (%)**	**Age m (SD)**	**Female n (%)**	**BD diagnosis n (%)BD-I BD-II BD-NOS**	**BPDSI mean (SD)**
PDQ-4+	111 (100)	46.3 (11.2)	73 (65.8)	62 (55.9) 44 (39.6) 5 (4.5)	–
BPD negative	56 (50.5)	48.4 (11)	36 (64.3)	35 (62.5) 19 (33.9) 2 (3.6)	–
BPD positive	55 (49.5)	44.2 (10.8)	37 (67.3)	27 (49.1) 25 (45.5) 3 (5.5)	–
Drop out	11	42.8 (9.2)	7 (63.6)	6 (54.6) 4 (36.4) 1 (9.1)	–
SCID-II	44 (100)	44.5 (11.3)	30 (68.2)	21 (47.7) 21 (47.7) 2 (4.6)	–
BPD negative	19 (43.2)	50.5 (8.2)	12 (63.2)	8 (42.1) 11 (57.9) 0 (0)	–
BPD positive	25 (56.8)	40.12 (11.4)	18 (72)	13 (52) 10 (40) 2 (8)	23.73 (7.2)
Drop out	12	42.7 (11.3)	9 (75)	7 (58.3) 4 (33.3) 1 (8.3)	21.82 (6.6)
STEPPS	13	37.8 (11.5)	9 (69.2)	6 (46.2) 6 (46.2) 1 (7.7)	25.5 (7.6)
Drop out	2	44 (14.1)	1 (50)	2 (100) 0 (0) 0 (0)	31.9 (2.1)
Missing data	2	29.5 (14.9)	2 (100)	1 (50) 0 (0) 1 (50)	26.1 (10.8)
Completers	9	38.2 (10.9)	6 (66.7)	3 (33.3) 6 (66.6) 0 (0)	23.3 (7.6)

*BPD, borderline personality disorder; BD, bipolar disorder; BD-I, bipolar I disorder; BD-II, bipolar II disorder; BD-NOS, bipolar disorder not otherwise specified; BPDSI, borderline personality disorder severity index*.

### Screening for PD According the PDQ-4+

In total 111 BD patients completed PDQ-4+, of whom 78 (70.3%) screened positive on at least one PD. Mean number of positive PD screening was 3.1. Most frequently patients screened positive on BPD (*n* = 55; 49.5%), avoidant PD (49.5%), obsessive compulsive PD (36.9%), and depressive PD (36.0%). Most frequent BPF reported on the self-report questionnaire (PDQ-4+) by the 111 BD patients were affective instability (73.9%), impulsivity and identity disturbance (both 53.2%), and fear of abandonment (48.6%). Impulsivity and anger outbursts were reported by 13.5% and 27.0%, respectively.

### Results SCID-II and BPDSI

In total 44 of the 55 patients who screened positive for BPD according to PDQ-4+ completed the BDP section of the SCID-II interview (Table 1). Most common borderline features were affective instability (79.5%), outburst of anger (63.6%), fear of abandonment (52.3%) and impulsivity (50%). Of these 44 patients, 23 (52.3%) had BPD according to SCID-II and another two had at least three BPF according to the definition of this study. Total mean score on severity index BPDSI (potential range 0–90) in this group (*n* = 25) was 23.72 (SD 7.22). Borderline features with highest mean BPDSI score were affective instability (mean = 5.21), emptiness (mean = 4.5), fear of abandonment (mean = 3.17) and identity (mean = 3.12).

### Treatment Effect

**Figure 1:**
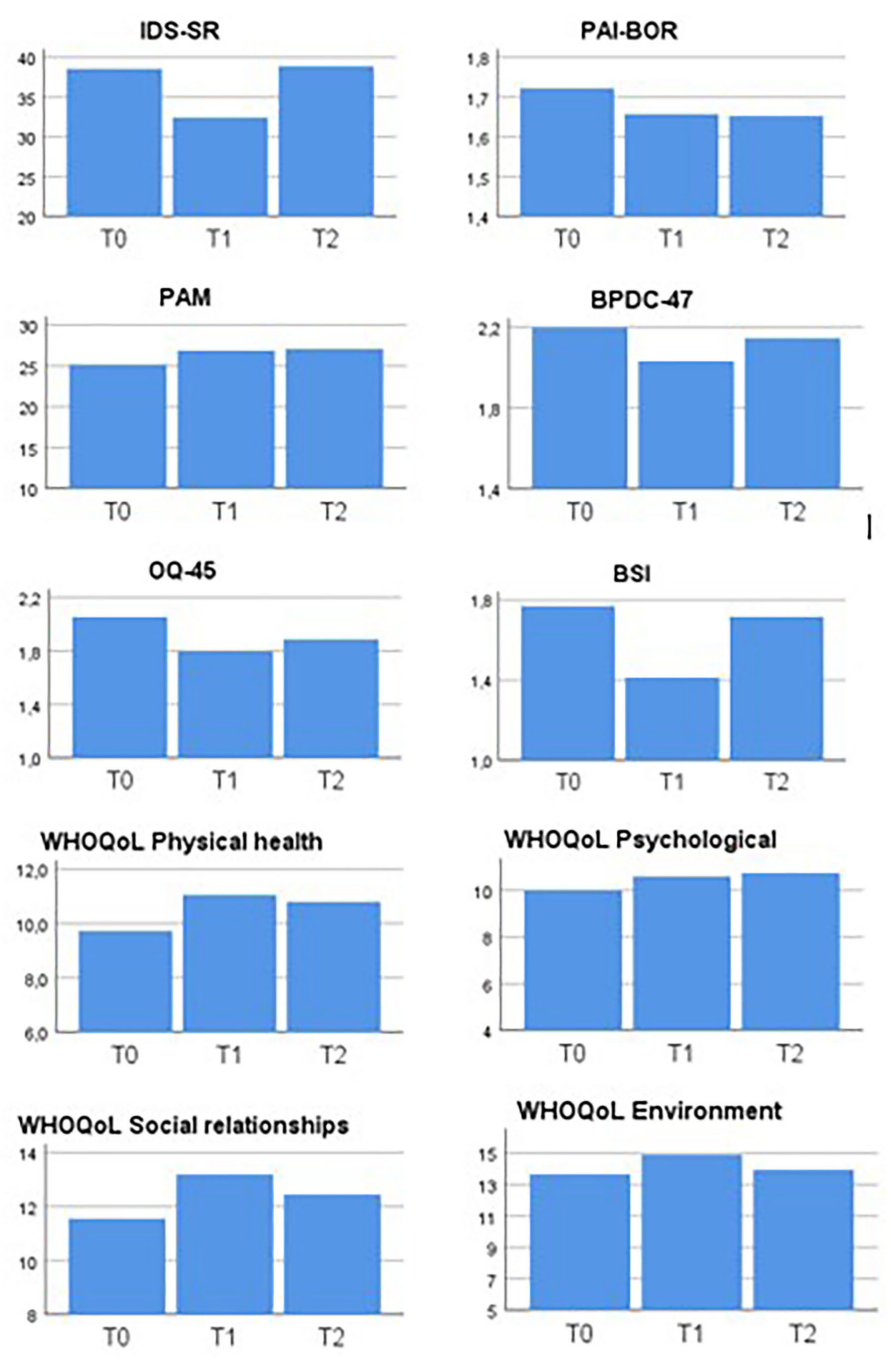
Scores of self-rated outcome questionnaires at baseline (T0), post treatment (T1), and 6-month follow-up (T2) in nine outpatients with bipolar disorder treated with STEPPS. IDS-SR, Inventory of Depressive Symptoms—Self Rated; PAI-BOR, Personality Assessment Inventory—Borderline Features; BPDC-47, Borderline Personality Disorder Checklist; PAM, Patient Activation Measure; BSI, Brief Symptom Inventory; OQ-45, Outcome Questionnaire; WHOQoL, World Health Organization Quality of Life short version.

Thirteen patients entered the open treatment study, which was embedded in regular STEPPS groups at the four sites. Two patients did not complete the treatment: one patient dropped out because of getting a new job and another because of feeling misplaced in a group treatment for borderline personality disorder. Another two had incomplete follow-up data. No patients dropped out because of developing a manic or depressive episode. Nine patients completed the treatment and study protocol. The scores of the self-rated outcome questionnaires at baseline (T0), post treatment (T1) and 6-month follow-up (T2) are summarized in Figure 1.

Descriptive statistics showed an improvement on all outcome measurements between baseline and post treatment measurement. In case of symptom inventories BSI, IDS-SR, BPDC-47 and on both measurements of Quality of life this positive effect disappeared on the 6-months follow up measurement. Improvement on PAM and PAI-BOR still were apparent on second follow-up measurement.

## Discussion

STEPPS was developed for improving emotion regulation in adult patients with borderline personality disorders (38), but could also be effective for other patient groups. A recent published pilot study (56) explored the effectiveness of STEPSS in adolescents with emotion regulation problems. Given the overlapping characteristics of BD and BPD including the significant impairment in emotion regulation strategies in comorbid BD and BPD (20), and the emotional instability of BD as such, we hypothesized that STEPPS would be suitable for patients with BD and some features of BPD. According to the STEPPS training manual the underlying assumption is that BPD is a disorder of emotion and behavior regulation and STEPPS learns participants specific emotion and behavior management skills. Since the BPF anger and impulsiveness as such are not included in the diagnostic criteria for BD, we considered that BD patients having these features could benefit most from STEPPS. Conversely, mood instability was excluded from our obligatory set of at least three BPF since this is a core symptom of BD. It was further hypothesized that STEPPS would not only improve its primary target of emotion regulation, but thereby would also improve the overall course of BD during and after the treatment. Half of the BD patients in our diagnostic study screened positive on BPD according to PDQ-4+, and half of these had a diagnosis of BPD after semi-structured interviewing. Reported prevalence of BPD in BD varies from 0.5 to 30% for BD-I and 12–23% for BD-II (21). Different studies (17, 57) conclude that ~20% of patients diagnosed with either BD or BPD also met criteria for the other diagnosis. Our study shows that the prevalence of BPD in BD patients depends highly on the instrument used, and that using a screener such as the PDQ4+ will result in many false positives. This confirms other studies that report low agreement between PDQ-4+ and diagnostic interviews (58). The diagnostic part of our study shows that BPF are widespread among patients with BD, and that anger outbursts and impulsivity are common. Since the STEPPS training focuses especially on these traits this supports our assumption that BD patients can befit from the treatment.

Our study was originally designed to evaluate the efficacy STEPPS in BD with BPF in a RCT (39). Due to much lower inclusion than expected the study design was subsequently redesigned as an open explorative study. Although that design and the limited number of participants prevent firm conclusions about the efficacy of the intervention, our findings give an indication that STEPPS may have a positive effect on illness symptoms as well as on patient empowerment and quality of life. This positive effect may be explained by the fact that the three most common BPF in our sample (affective instability, anger outbursts, and impulsivity) are especially targeted in the STEPPS training. Still, as seen often in psychotherapeutic interventions, the effect faded at 6-month follow-up.

Only nine patients completed the treatment part of the study. Several reasons can be hypothesized for this notable gap between the intended sample size and the low inclusion rate. We recruited patients from four different non-academic outpatient clinics. Although we expected a broad interest among clinicians as well as patients for this psychotherapeutic intervention, only 111 patients showed interest in the study and were willing to participate in the first diagnostic part of the study, completing the PDQ-4+, and if screened positive for BPD, receiving a semi-structured diagnostic interview. An important reason for not further participating in the subsequent treatment study, was that a treatment duration of 20 weeks and the additional follow-up of 18 months including continuous mood monitoring was considered too intensive and too long. Furthermore, some participants stated that the association of STEPPS with “borderline” had an aversive effect, which may have caused drop-out before the treatment stage. Moreover, the intervention was intendedly embedded in the regular STEPPS programs of the outpatient clinics, so not exclusively for the patients with BD in this study. Stigma is a well-known phenomenon in patients with BPD. In a study that measured self-stigma by self-report questionnaires, women with BPD showed higher rates of self-stigma than women with social phobia (59). Also perception of health professionals is laden with stigma toward diagnosis of BPD (60–62). One study showed that the description of a patient with diagnosis of BPD attracted more negative response from a sample of qualified mental health nursing staff compared with descriptions of patients with a diagnosis of depression or schizophrenia (63). Sympathy and optimism was rated less toward patients with a diagnosis of BPD, and personal experience with patients with BPD was rated as more negative than experience with patients with diagnosis of depression or schizophrenia (63). In line with these findings, negative stigma toward BPD could explain our disappointing inclusion in the treatment part of the study.

Our study raises the question whether a treatment primarily developed for one diagnostic group (such as personality disorder) can be applied to another patient population (such as mood disorder), even with a rationale as described. We assumed that embedding the study protocol in ongoing STEPPS programs would increase the feasibility of this treatment option in routine clinical practice, but this may have been counterproductive. In case of STEPPS, primarily designed for BPD patients, we therefore recommend to adapt the training manual and materials for participants before being applied to BD patients, and forming a group specifically for BD. Avoiding the term “borderline,” one could describe that BD includes problems with emotion regulation and that these are the main focus of STEPPS. In the context of research this would imply that inclusion should not be based on “borderline personality features” but instead focus on emotion regulation.

Our study has several limitations. First, the sample size of the treatment part was small for reasons explained above. Second, even if more patients had participated in the adapted study design, the methodological nature of this open non-controlled study prohibits conclusions of causal relations between the treatment and possible effects, since only a RCT can show the effectiveness of STEPPS in BD.

Despite these limitations, we conclude that a modified version of STEPPS could be a promising addition to the evolving field of psychological treatments for patients with BD.

## Data Availability Statement

The original contributions presented in the study are included in the article/supplementary material, further inquiries can be directed to the corresponding author.

## Ethics Statement

This study was reviewed and approved by the Ethics Committee of the VU University Medical Center in Amsterdam, The Netherlands (registration number: 2012/470). It is registered in www.trialregister.nl (trial ID: NTR4016). The patients/participants provided their written informed consent to participate in this study.

## Author Contributions

GR, NW, ER, MC, and RK: conception and design, and/or acquisition of data, and/or analysis and interpretation of data, drafting the article or revising it critically for important intellectual content, and give final approval of the version to be submitted and any revised version. All authors contributed to the article and approved the submitted version.

## Funding

This research was supported by the Netherlands Organization for Scientific Research (NWO). The supporters had no role in the design, analysis, interpretation, or publication of this study.

## Conflict of Interest

The authors declare that the research was conducted in the absence of any commercial or financial relationships that could be construed as a potential conflict of interest.

## Publisher's Note

All claims expressed in this article are solely those of the authors and do not necessarily represent those of their affiliated organizations, or those of the publisher, the editors and the reviewers. Any product that may be evaluated in this article, or claim that may be made by its manufacturer, is not guaranteed or endorsed by the publisher.

## Abbreviations

BD: Bipolar disorder
BPD: Borderline Personality Disorder
BPF: Borderline Personality Features
RCT: Randomized Controlled Trial
STEPPS: Systems Training for Emotional Predictability and Problem Solving.
